# Non-equilibrium dynamics and floral trait interactions shape extant angiosperm diversity

**DOI:** 10.1098/rspb.2015.2304

**Published:** 2016-05-11

**Authors:** Brian C. O'Meara, Stacey D. Smith, W. Scott Armbruster, Lawrence D. Harder, Christopher R. Hardy, Lena C. Hileman, Larry Hufford, Amy Litt, Susana Magallón, Stephen A. Smith, Peter F. Stevens, Charles B. Fenster, Pamela K. Diggle

**Affiliations:** 1 Department of Ecology and Evolutionary Biology, University of Tennessee, Knoxville, TN 37996, USA; 2 Department of Ecology and Evolutionary Biology, University of Colorado, Boulder, CO 80309, USA; 3 School of Biological Sciences, University of Portsmouth, Portsmouth PO1 2DY, UK; 4 Institute of Arctic Biology, University of Alaska, Fairbanks, AK 99775, USA; 5 Department of Biology, Norwegian University of Science and Technology, 7491 Trondheim, Norway; 6 Department of Biological Sciences, University of Calgary, Calgary, Alberta, Canada T2N 1N4; 7 Biology Department, Millersville University, Millersville, PA 17551, USA; 8 Ecology and Evolutionary Biology, University of Kansas, Lawrence, KS 66045, USA; 9 School of Biological Sciences, Washington State University, Pullman, WA 99164, USA; 10 Department of Botany and Plant Sciences, University of California, Riverside, CA 92521, USA; 11 The New York Botanical Garden, Bronx, NY 10459, USA; 12 Instituto de Biología, Universidad Nacional Autónoma de México, México DF 04510, México; 13 Ecology and Evolutionary Biology, University of Michigan, Ann Arbor, MI 48109, USA; 14 Missouri Botanical Garden, St Louis, MO 63166, USA; 15 Department of Biology and Microbiology, South Dakota State University, Brookings, SD 57007, USA; 16 Department of Ecology and Evolutionary Biology, University of Connecticut, Storrs, CT 06269-3043, USA

**Keywords:** diversification, floral evolution, macroevolution, non-equilibrium, flower symmetry, pollination

## Abstract

Why are some traits and trait combinations exceptionally common across the tree of life, whereas others are vanishingly rare? The distribution of trait diversity across a clade at any time depends on the ancestral state of the clade, the rate at which new phenotypes evolve, the differences in speciation and extinction rates across lineages, and whether an equilibrium has been reached. Here we examine the role of transition rates, differential diversification (speciation minus extinction) and non-equilibrium dynamics on the evolutionary history of angiosperms, a clade well known for the abundance of some trait combinations and the rarity of others. Our analysis reveals that three character states (corolla present, bilateral symmetry, reduced stamen number) act synergistically as a key innovation, doubling diversification rates for lineages in which this combination occurs. However, this combination is currently less common than predicted at equilibrium because the individual characters evolve infrequently. Simulations suggest that angiosperms will remain far from the equilibrium frequencies of character states well into the future. Such non-equilibrium dynamics may be common when major innovations evolve rarely, allowing lineages with ancestral forms to persist, and even outnumber those with diversification-enhancing states, for tens of millions of years.

## 1. Introduction

Disparity in the numbers of taxa with different traits is a hallmark of biodiversity. Explanations for the distribution of phenotypes across the Tree of Life variously emphasize evolutionary processes acting both above and below the species level [1]. For example, as a result of intraspecific processes such as mutation, selection and genetic drift, some traits may be gained more often than lost, and the ensuing directionality in transitions should lead to more taxa having those traits [2–4]. Above the species level, differential speciation and/or extinction across lineages can also shape the distribution of phenotypic diversity [5,6]. For instance, traits that act as key innovations are expected to become common across the tree [7], whereas those that act as evolutionary dead-ends will continually be pruned from the tree [8]. Together, these long-term rates of character change, speciation and extinction establish an expected equilibrium frequency of different phenotypes [9]. However, because the effects of these underlying processes accumulate gradually over long evolutionary timescales, non-equilibrium dynamics may also be important. For instance, even traits leading to rapid lineage diversification may be rare in a clade if they evolved only recently. Despite the growth of comparative methods for the analysis of trait evolution and diversification, the extent of non-equilibrium dynamics in natural systems remains largely unexplored [10].

Here we examine the interplay of trait transition rates, differential diversification and non-equilibrium dynamics in the history of angiosperms. This approximately 140 million year old clade [11] comprises at least 250 000 species known for both their amazing variety of forms and the markedly uneven distribution of this phenotypic variation across taxa. For example, some floral morphologies appear in many families and species, whereas other forms characterize only a few taxa [12]. Stebbins [13] attributed the abundance of taxa with particular floral phenotypes to directional transitions generated by natural selection, and moreover, he suggested that variation in functional interactions among traits, rather than in the traits themselves, leads to differences in fitness. However, a skewed distribution of floral forms with some common and others rare could also arise from differential diversification, for example, if certain forms promote speciation more than others. This differential diversification could be linked to individual floral traits, e.g. floral symmetry [14], or, if functional interactions are key, to combinations of characters, e.g. stamen number and symmetry together [15]. Distinguishing between these possible explanations for the distribution of floral morphologies across angiosperms requires simultaneous estimation of lineage diversification and trait transitions [2,9]. Although previous studies have examined the patterns of trait evolution across the angiosperms [4,14,16,17], none have quantified the relative importance of directional trait transitions and differential diversification in the present-day distribution of floral traits and of trait combinations, or assessed whether this distribution represents an evolutionary equilibrium.

We focus on the evolution of a suite of six floral traits, including aspects of merosity, fusion and symmetry, that were originally considered by Stebbins [13] in his angiosperm-wide analysis. These features vary dramatically across taxa [18,19] and are strongly associated with floral function [20,21]. Given their role in interactions with pollinators, these traits are probably under strong selection, with adaptive changes spreading to fixation whenever they arise. In addition, differences in the interaction between flowers and pollinators may lead to reproductive isolation and diversification [22,23]. Plant–pollinator interactions often generate selection for suites of floral traits [24], supporting Stebbins' notion that trait combinations are key to understanding plant adaptation and speciation. Collectively, these prior studies suggest that directional transitions and differential diversification acting on interacting suites of floral traits may have played a significant role during the angiosperm radiation. To test this question, we amassed trait data and phylogenetic information for a large and random sample of angiosperms and developed scripts for model fitting with multiple characters and character combinations with existing software. With this approach, we consider to what extent the present-day frequency of the six target traits is owing to directional transitions and differential diversification. We also ask if directionality and differential diversification hinge on single character states or the states of multiple characters. Finally, using stochastic simulations based on these results, we examine whether the distribution of angiosperm diversity has reached equilibrium, i.e. whether the frequencies of taxa with different floral trait combinations match predictions based on rates of trait transitions and species diversification. While evolutionary lags are predicted to shape the course of adaptive radiations [10], it is unknown if such non-equilibrium dynamics are important on such a deep evolutionary timescale.

## 2. Methods

### (a) Phylogenetic inference

Five hundred angiosperm species were selected from GenBank for inclusion in the dataset. Available software for joint estimation of transition rates and state-dependent diversification, Diversitree [25], allows for incomplete taxon sampling (i.e. not all extant species present in the phylogeny), but the subsampling is assumed to be random across clades (i.e. clades are sampled in proportion to their species richness, though unequal sampling based on traits can be incorporated) [26]. We thus implemented a stratified two-step procedure to create a random sample of species that satisfies this criterion. The stratified random sampling procedure first calculated the expected number of species sampled from each angiosperm family given the size of the family; larger families would accordingly be represented by more species. If the expected number was less than one (families of fewer than 500 species), we used dynamic rounding to convert the fraction to an integer. That is, if a random variate between 0 and 1 was larger than the expected fraction, one species was chosen, and otherwise, none was selected. Second, among the species in GenBank with sufficient sequence data (minimally ITS), we randomly selected the expected number of species from each family. For example, a family represented by 1% of all extant angiosperms would comprise 1% (or 5 species) of our 500 species dataset, and we randomly chose five species from the family among those on GenBank that minimally had ITS sequences. We compiled sequences for six other loci in addition to ITS (*rbcL*, *matK*, 28S, 18S, *trnK*, *atpB*) and built our final supermatrix with the PHLAWD pipeline [27]. We chose a set of 28 additional angiosperms (e.g. *Amborella*, *Austrobaileya*) for localizing fossil calibrations (electronic supplementary material, table S1), and we selected 44 gymnosperms to serve as outgroups. We inferred the phylogeny in RAxML v. 7.2.3 [28] using a GTR + gamma model of sequence evolution, partitioned by gene, and a family-level constraint tree based on well-established relationships (http://www.mobot.org/MOBOT/research/APweb/, accessed on 8 January 2009). We employed a constraint tree, because the goal of this analysis was not to re-infer angiosperm relationships [29–31], but to obtain relative branch length estimates for the sampled tips. The RAxML tree was time-calibrated using penalized likelihood in TreePL [32] with 40 fossil calibration points (electronic supplementary material, table S1). The outgroups and additional angiosperms were pruned from the tree before analysis to maintain the random sampling design.

### (b) Character selection and scoring

Based on Stebbins' 1951 analysis of angiosperm diversity [13], we targeted six binary floral characters: corolla (petals) presence, fusion of the perianth (calyx and corolla or tepals), flower symmetry, stamen number relative to perianth parts, carpel fusion and ovary position. Changes in perianth morphology and relative stamen number alter interactions with pollinators and thereby affect the accuracy and efficiency of pollen transfer [20,21,33,34]. Carpel fusion influences the intensity of competition among pollen tubes for access to ovules and allows more even distribution of pollen tubes among carpels, which can enhance offspring quality and quantity [35,36]. Finally, inferior ovaries may protect ovules from probing insects [37] and facilitate coevolution with ovipositing insects [38] relative to superior ovaries. We scored all 500 species in the phylogeny for the six characters based on floras, monographs and species descriptions (e.g. [39,40], see the electronic supplementary material, table S2 for more detail). The final dataset (phylogeny and character matrix; Dryad http://dx.doi.org/10.5061/dryad.486c0) was reduced to 464 angiosperm species, because some states could not be scored for some taxa (e.g. perianth symmetry cannot be scored for species without a perianth).

### (c) Joint analyses of transition and diversification rates

**Figure 1.**
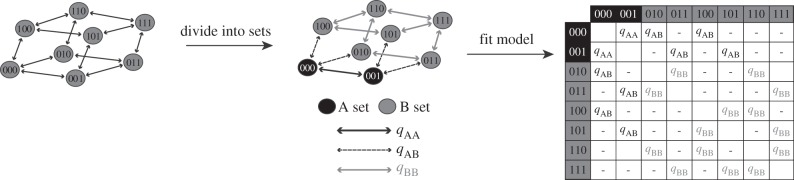
Simplification of MuSSE model by partitioning into sets. The diagram depicts a network of eight states, which are combinations of three binary (0/1) characters. Thus, ‘000’ is the combination with state 0 for each of the three characters. Each combination is connected to three other combinations that differ by just one state. A full MuSSE analysis would involve estimating transition rates (black arrows) along each of the edges of this network as well as speciation and extinction rates (not shown) for each combination. We simplified this network by dividing it into two sets (e.g. the black ‘A’ set and the grey ‘B’ set in the middle). We can then consider rates of transition within the sets (*q*_AA_, *q*_BB_) and between the sets (here, *q*_AB_ = *q*_BA_). This can be represented as a matrix (right), and if we assume a single rate within each set plus a rate of moving between sets, the model includes just three transition rate parameters. We could also assume a single speciation and an extinction rate for each set (not shown), which would bring the model to seven parameters. These assumptions can be relaxed to create additional more complex models, but which are still less parameter-rich than the full model.

We employed SSE methods [9,26] for jointly estimating transition rates among character states, character state combinations and diversification rates within those combinations. Multistate SSE methods (MuSSE [25]) can handle a single multistate character (with states 0, 1, 2, etc.) or a set of binary characters. The latter can be done by converting the set of binary characters to a single multistate character. For example, for a set of three characters, state combination 000 becomes state 0, combination 001 becomes state 1, 010 becomes state 2 and so forth, giving eight states in total (figure 1). In our case, with six binary floral characters, there are 2^6^ (or 64) possible combinations, which can be coded as a single character with 64 states. The full MuSSE for a 64-state character would have 512 free parameters (384 transition rates, 64 speciation rates and 64 extinction rates), which would be challenging to estimate, even with a complete sampling of all angiosperms. Therefore, we created a set of scripts to fit a series of simplified models that essentially group the states (and thus the rates) while still allowing us to test hypotheses about directional transition and/or differential diversification (see full workflow in the associated Dryad package: http://dx.doi.org/10.5061/dryad.486c0). As an example, returning to the three character network in figure 1, we could group the states into two sets (000 + 001 versus the rest) and ask how having a zero for the first two character states affects diversification rates. We would estimate just two pairs of state-dependent speciation and extinction rates (one for the A set and one for B set) instead of the eight required for the full model (not shown in figure 1). We also need to estimate transition rates, but could assume that transitions within each set has a single rate (*q*_AA_ or *q*_BB_), and there is just one rate for moving between the sets (*q*_AB_; figure 1). Thus, we have three rate parameters to estimate instead of the 12 in the original full model. We could relax these constraints in order to test for directional transitions, i.e. allow transitions from A into B to occur at a different rate than B into A, which would add one additional parameter. This approach of grouping states into sets results in a tremendous reduction in the number of model parameters as the state space grows. Even with the most complex model, having directional transitions between A and B and different rates of diversification in A and B, we have only eight parameters to estimate (two speciation rates, two extinction rates, four transition rates) compared with the 512 in the full model.

In order to test for directional transitions and differential diversification associated with each character state and character state combination, we conducted this subsetting procedure across all possible bipartitions (division into two sets) of the network of 64 combinations. For individual characters, this division split the network into two equal halves, namely all the combinations with a zero for the character and all of those with a one (0***** versus 1***** where the * may be any state for any of the other characters). Bipartitions can also be as small as a single combination versus the rest (e.g. 000111 versus all other combinations). Through this procedure, then, we examine all characters individually and in all possible combinations with the other characters. For each of these bipartitions, we then tested a set of models that varied in constraints on transition and diversification rates. In total, we considered 30 models, all combinations of five transition rate models (electronic supplementary material, table S3) and six diversification models (electronic supplementary material, table S4), for each bipartition. We used maximum-likelihood MuSSE functions implemented in the R package Diversitree [25] to estimate diversification and transition rates for each model, to calculate the Akaike information criterion (AIC) scores, and to infer ancestral states across the phylogeny. Rather than assume an equilibrium root state or otherwise infer it, we assumed that lineages began with the ancestral states for each character (electronic supplementary material, table S2), which follows the coding by Stebbins' [13] and more recent comparative studies [41,42]. The version of Diversitree used allows specification of sampling by state: absent other information, we assumed the sampling within each state was the same (464 out of 250 000 angiosperm species). Note that in grouping states into sets, we did not alter the underlying data (the six characters), so likelihoods are comparable between all models and bipartitions.

### (d) Stochastic simulations

We conducted two sets of simulation analyses to interrogate the findings from maximum-likelihood model fitting. The first set of simulations assessed how the level of sampling (464 of an estimated 250 000 angiosperms) might affect the reliability of our conclusions. To do this, we conducted parametric bootstrapping under the inferred model. We used model-averaged values for speciation, extinction and transition rates, weighted by the AIC scores of the models, from the MuSSE analysis. We simulated phylogenies under this model using the SimulateHisse function in the R package HiSSE [43], starting with a single lineage and proceeding for 136 million years (the approximate crown group age of angiosperms based on recent analyses and pollen fossils [44,45]). At every instant of time, any species could speciate, go extinct or change state. This procedure made trees of thousands to hundreds of thousands of species, which we then randomly pruned to 464 species. We re-estimated the 30 diversification and transition models that were fitted to the original dataset, focusing on a set of combinations that were found to have higher diversification in our MuSSE analysis. Finally, we compared the model-averaged rates from these simulated replicates with those estimated from the original data.

The second set of stochastic simulations estimated the future approach towards an equilibrium distribution of trait combination frequencies based on the inferred transition and diversification rates. The SSE methods do not necessarily assume equilibrium, and thus the observed distribution of current states may differ from that predicted under the best model at equilibrium [8,46]. We again used the model-averaged rates from the original analysis and simulated evolution for 151 million years from the original ancestral state (i.e. 15 million years into the future). In these simulations, we tracked the proportion of species with each trait combination at 1 million year intervals, but not the tree structure itself, unlike the first simulation (this makes the computation far more efficient). To test for non-equilibrium, we compared the proportions of taxa with each combination of the three key characters (corolla presence, floral symmetry and stamen number; see Results) across the simulations to the proportions expected at equilibrium. We calculated these equilibrium values from the model-averaged diversification and transition rates following Maddison *et al*. [9] using stationary.freq.classe() in Diversitree. The scripts for running both of these sets of simulations are available as part of the Dryad package containing the dataset.

## 3. Results

### (a) Distribution of trait combinations

Our sample of 464 species was spread across 134 angiosperm families. Across these species, the character–state combinations exhibited a wide range of frequencies (electronic supplementary material, figure S1). Thirty-one, or 48%, of the 64 possible combinations are not present in any sampled species, suggesting that much of phenotype space is unoccupied. Within the occupied space, over half of all sampled species exhibit the five most common combinations, which all share the presence of a corolla, few stamens, and fused carpels. This skewed distribution of character combinations contrasts sharply with the distribution obtained by randomly sampling the combination space (grey lines, electronic supplementary material, figure S1). The concentration of angiosperm diversity in certain regions of floral phenotype space has been considered qualitatively and even quantitatively in previous studies [12,13], but this is, to our knowledge, the first demonstration based on a taxonomically stratified random sample across the angiosperms at the species level. We expect that the remaining sampling bias is due to which species within each family are found in GenBank and available for scoring.

### (b) Differential transition and diversification rates across character combinations

We examined a large number of models to identify character–state combinations associated with directional transitions and/or differential diversification. Given the 728 (or 3^6^ – 1) possible bipartitions of the 64-state network into sets of character–state combinations and the 30 diversification/transition models, the total possible number of models to examine is 21 840. However, many character–state combinations are not represented by any species, and thus could not constitute a viable set, leaving 19 657 models. Despite this large model space, over 99% of the Akaike weight was concentrated in just 10 models. All 10 of these models incorporate differential diversification and include one or more of the three character states: corolla present, bilateral symmetry and reduced stamen number (table 1). According to the best-fitting model (lowest AIC score), lineages with this combination of characters diversified roughly twice as fast as lineages with any other character–state combination (table 1, compare *r*_F_ with *r*_N_). In contrast to the diversification rates, which varied based on floral state combinations, we found little evidence for strongly asymmetric trait transition rates that could lead to biases in state frequencies. The best-fitting model for the set comprising bilaterally symmetrical corollas with reduced stamen number included equal transition rates between the sets (table 1). Furthermore, models with differential transition rates accounted for only 29.8% of the AIC weight across all of the analyses. Thus, differences in gain and loss rates (directional transitions) appear to have had a limited influence on the overall distribution of trait combinations across angiosperms.

**Table 1.** Top 10 models (ranked by Akaike weight) from the maximum-likelihood analysis of focal areas. (The focal area is described by a string in which 0 and 1 indicate the ancestral or derived states, respectively, for each of the six characters (electronic supplementary material, table S1) and ‘asterisk’ indicates that the state for that character could be either 0 or 1. The only characters identified by the models as part of focal area are corolla presence (character 1: 0, present; 1 ,absent), floral symmetry (character 3: 0, radial; 1, bilateral) and stamen number (character 4: 0, more than twice as many as merosity; 1, equal or fewer). For example, the focal area in the top model (first row, 0*11**) comprises all taxa with corolla present, bilateral symmetry and few stamens, regardless of the states of the other three characters. Model parameters include transition rates within and between focal (F) and non-focal (N) areas (*q*_FF_, *q*_NN,_ *q*_FN_, *q*_NF_) and diversification parameters (*r*_F_, *r*_N_). Complete description of models for transitions and diversification are presented in tables S3 and S4).)

Akaike weight	cumulative Akaike weight	focal	diversification	*r*_F_	*r*_N_	transitions	*q*_FN_	*q*_NF_	*q*_NN_	*q*_FF_
0.274	0.274	0*11**	free	0.064	0.038	equal	0.001	0.001	0.001	0.001
0.245	0.519	**1***	free	0.057	0.037	free	0.003	0.001	0.002	0.000
0.202	0.721	**1***	free	0.052	0.036	equal	0.001	0.001	0.001	0.001
0.128	0.849	**11**	free	0.059	0.038	equal	0.001	0.001	0.001	0.001
0.090	0.939	0*1***	free	0.058	0.038	equal	0.001	0.001	0.001	0.001
0.024	0.963	**1***	free	0.055	0.034	outflow	0.003	0.001	0.001	0.001
0.014	0.977	0*11**	free	0.060	0.035	outflow	0.001	0.001	0.001	0.001
0.009	0.985	**1***	two/one	0.068	0.030	free	0.005	0.001	0.002	0.000
0.003	0.988	0*1***	free	0.059	0.035	outflow	0.002	0.001	0.001	0.001
0.003	0.991	0*11**	free	0.050	0.037	free	0.001	0.003	0.002	0.000

**Figure 2.**
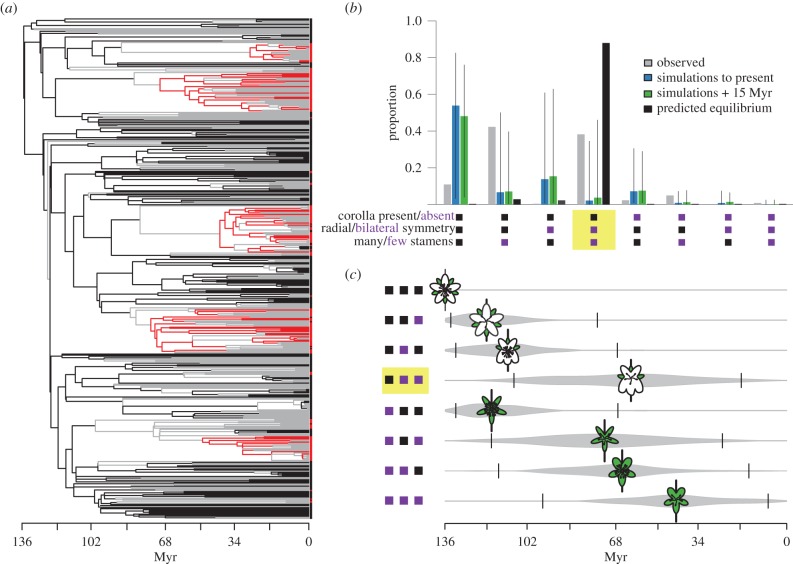
Non-equilibrium dynamics and the evolution of the floral trait combinations. (*a*) Maximum-likelihood reconstructions of the history of the three key character combination using model-averaged rates from the MuSSE analysis. Repeated origins of the key combination are shown in red. All other combinations are shown in black or grey lines, with the latter indicating uncertainty in the reconstruction of the subtending node. (*b*) The proportion of taxa with each possible combination of states for the three characters that comprise the key combination (corolla presence/absence, symmetry, and relative stamen number) in the observed (empirical) dataset (grey), in stochastic simulations to the present (blue), in simulations extending 15 Myr into the future (green), and at equilibrium (black). Ancestral states are shown in black and derived states in purple (see also the electronic supplementary material, figure S1), and the key combination is highlighted in yellow. (*c*) Mean (±95% CI) time of appearance of each of the eight trait combinations defined in (*b*) during the course of simulations. Flower cartoons show states of perianth (white, corolla; green, calyx), symmetry and stamen number.

Despite the weak direct effect of differences in gain and loss rates on the frequencies of trait combinations, the low overall magnitude of all transition rates compared with diversification rates (table 1) could profoundly affect the distribution of trait combinations through non-equilibrium dynamics. Given the estimated rates, we calculate that, at equilibrium, 90% of angiosperms should possess the key set of states associated with higher diversification, namely bilaterally symmetrical corollas and few stamens. By contrast, only 38% of the species in our sample belong to that set. This marked difference between the equilibrium expectation and the observed frequency could arise because rare transitions between character states limit the potential for lineages to assemble the three elements of the key combination. Comparison of the transition rates and diversification rates (table 1) is consistent with this explanation. In the best-fitting model, the diversification rate of lineages with the ancestral state is 38 times higher than the rate of transitions away from that state, suggesting an average of 38 net diversification (speciation minus extinction) events for every transition event. The ratios are similar for other character state combinations. These low transition rates relative to diversification rates are also consistent with large clades sharing the same state (figure 2*a*) and with a higher number of species retaining the ancestral state than predicted at equilibrium (corolla present, radial symmetry, many stamens; figure 2*b*).

### (c) Stochastic simulations of angiosperm evolution

Our simulations illustrate that the preceding conclusions about directional transitions and state-dependent diversification are probably not an artefact of sampling intensity. Using model-averaged rates from the likelihood model fitting, we completed 136 million year simulations for 22 of the 50 lineages that we initiated. The resulting 22 trees contained from 2673 to 186 550 species (owing to stochasticity), which were randomly subsampled to 464 species (the size of our original dataset) before model fitting. As in the original dataset, the simulations supported elevated diversification for lineages with the key set of states (bilaterally symmetric corollas with reduced stamens, electronic supplementary material, figure S2). For the three key characters, diversification rates were, on average, 30% higher in the set with bilaterally symmetric corollas with reduced stamens than in the other set across all of the simulated datasets (electronic supplementary material, table S5). Moreover, the original rates fell within the range of the simulations for both sets of diversification and transition rate parameters (electronic supplementary material, figure S2). Thus, detection of elevated diversification in lineages with bilaterally symmetric corollas and reduced stamens is robust to the sampling intensity employed in our analysis.

An independent set of stochastic simulations explored the consequences of relatively low transition rates for non-equilibrium dynamics. Estimates of the equilibrium frequency across trait combinations predict that most angiosperm species should eventually possess bilaterally symmetric corollas with reduced stamens, compared with the 38% at present. Our stochastic simulations showed an even lower proportion of species in this set after 136 million years of evolution (7%, 95% confidence interval (CI) 0–35%: figure 2*b*). The wide CIs around the mean from these simulations reflects the stochastic and contingent nature of evolutionary processes [47]; a variety of outcomes are possible even when the starting point and parameters are the same. Despite the range of outcomes from the simulations, none of the simulations produced the high proportion of species with the key set expected at equilibrium (90%). At the same time, the proportion of species with the ancestral state (radially symmetric corollas with many stamens) is much higher in the simulations (53%, 95% CI: 3–84%) than predicted at equilibrium (0.2%), consistent with a long delay in evolving away from the ancestral state. This gap between the equilibrium expectations and the simulated proportions (as well as the observed proportions) supports the hypothesis that low transition rates relative to diversification rates have limited the rate of approach to equilibrium. Continuation of the simulations for an additional 15 million years to extrapolate future angiosperm evolution towards the equilibrium raised the frequency of lineages with the key set of character states to a mean of 10% (95% CI: 0–46%), but it still remained far below the expected equilibrium.

## 4. Discussion

Given the critical role of flowers in sexual reproduction, their morphology should be subject to strong selection [23,24]. Indeed, Stebbins [13] largely attributed the highly skewed pattern of floral trait combinations across the angiosperms (electronic supplementary material, figure S1) to strong natural selection leading to the fixation of particular combinations of characters. Over time, these directional transitions towards adaptive combinations would lead to the prevalence of some phenotypes and the rarity or complete absence of others. He recognized that genetic factors, such as pleiotropy [48] or genetic architectures which favour loss over gain [49], could also contribute to evolutionary trends in angiosperm morphology. Subsequent studies have reported directional trends for the evolution of many complex traits [50,51], including floral traits such as symmetry and colour [4,46] as well as the ability of natural selection to act on multiple floral trait combinations [52]. However, our study finds that directionality in rates of evolution has had a limited impact in determining the overall frequency of phenotypes for the six traits among angiosperms. The best-fitting model assumed equal rates of gain and loss of character states, and over all models examined, models with biased transitions received less than 30% of the AIC weight. This contrast between our study and previous studies that highlighted directionality is probably owing to our application of SSE methods to a random sample of angiosperms. These methods allow for the possibility that differences in diversification rates, in addition to transition rate biases, could contribute to patterns of trait variation in extant taxa [2], and thus do not presuppose a conclusion of directional transitions to account for unequal numbers of taxa in different states [3].

In contrast to the limited effect of directional transitions, our analysis identified differential diversification as an important component of the best-fitting models. Specifically, increased diversification is strongly associated with three key character states: corolla present, bilateral symmetry and few stamens. These floral features facilitate animal pollination, and their well-studied effects on plant–pollinator interactions are consistent with a role in shaping patterns of diversification. Bilateral floral symmetry is frequently proposed as a key innovation [14,53] and in our models, it affects diversification rates most consistently, occurring in all of the top 10 models. Critically, however, symmetry does not operate in isolation to influence diversification. The inclusion of two additional traits, corolla presence and stamen number, in the top models demonstrates that the positive effect of symmetry hinges on the state of other floral characters. Ecological studies show that both bilateral symmetry and reduced stamen number increase precision of pollen placement and facilitate specialization for particular pollinators [54–56]. Bilateral floral symmetry modulates pollen placement by constraining the approach of pollinators and positioning them with respect to the stamens [55]. Reductions in stamen number increase precision by promoting correspondence between the positions of pollen placement and stigma contact [54,56–58]. The inclusion of corolla presence among the key features suggests that bilateral symmetry is most important in flowers that have a perianth which includes a corolla, or that having a corolla provides greater flexibility of function for the calyx. Together, the potential for both more specialized and more precise interactions with pollinators may increase opportunities for prezygotic isolation and thus speciation in lineages with bilaterally symmetrical corollas and few stamens [14,23,59,60], although lower extinction rates may also play a role [60]. The many origins of this combination across the phylogeny (figure 2*a*) suggest that its association with diversification is not spurious [61,62], or limited to a single major clade, but instead that it is a key innovation that has arisen convergently in many angiosperm lineages.

If increased diversification owing to the key set of character states fully explained the pattern of trait disparity fitted by the models, the trait combination with greatest diversification rate should be most common, as is expected at equilibrium. By contrast, we estimate that this set is found in less than 40% of extant species. Our stochastic simulations suggest that this discrepancy is owing to the long time required for the evolutionary assembly of all three character states. In these simulations, a median of 11 million years (95% CI 2.0–37.4) elapsed before fixation of any trait combination other than the ancestral combination, and 71.5 million years (33.3–109.35) elapsed before the first origin of the three character combination with the highest diversification rate (figure 2*a,c*). These intervals are concordant with the fossil record, as flowers with reduced stamen number appeared within 20 million years after the origin of angiosperms, followed by bilaterally symmetric flowers roughly 44 million years later [63]. The number of angiosperms with the ancestral states continues to decline towards the present, whereas the number of taxa with the fastest diversifying combination of derived states continues to rise (figure 2*b*). Our simulations suggest that the approach to the equilibrium predicted by the model will be exceptionally gradual, as an additional 15 million years of evolution produced only a 1.5% closer approximation of the equilibrium frequencies. Thus, although the possession of bilaterally symmetrical corollas and few stamens clearly promotes diversification once it has evolved, the relative rarity of necessary transitions causes a lingering influence of the initial state of the common ancestor on current trait diversity. Given the slow pace of the approach to equilibrium, major global events (such as mass extinctions) that could alter the course of angiosperm evolution are likely to occur before the predicted frequencies are reached.

This inferred history of six functional floral traits reveals two general features that probably dominate the dynamics of trait evolution in many clades of organisms, and hence strongly influence the distribution of contemporary diversity. First, traits and trait combinations that experience low transition rates are likely to remain far from the expected equilibrium frequencies over long evolutionary timespans. This means that even character states that strongly promote lineage diversification may not be associated with high species richness at any instant, underscoring the importance of the timing of the ‘arrival of the fittest’ [64]. Second, many traits, like the features of floral organs, interact in organismal function and their effects, whether on the fitness of individuals in populations or on the success of lineages, depend on the state of other traits. Incorporating such context-dependence introduces additional complexity into studies of macroevolutionary patterns. Nevertheless, consideration of trait interactions in an organismal and functional framework will lead to a richer and more realistic picture of the evolutionary process and a stronger connection with microevolutionary studies, which have long emphasized synergistic effects [65,66].

As researchers move towards diversification analyses of multiple characters [67,68], we anticipate increasing interest in using approaches such as ours that directly address the potentially synergistic interactions between characters [15]. In this context, it is important to discuss the potential limitations of our analysis and of SSE methods in general. First, we assumed the tree and states were known with certainty, though relaxing these assumptions are possible. Second, SSE methods have been found to choose a trait-dependent diversification model when traits are simulated under a diversification-independent model and the tree comes from an unknown empirical process [69]. This reflects a well-known statistical issue: rejecting a null does not mean that the alternate model is correct. Here, we used multimodel inference rather than null hypothesis rejection, with a focus on the parameter values. This incorporates uncertainty in the models and focuses on the biological processes, rather than rejecting an unrealistic null. However, even though we used a variety of models, they were individually simple: there was some heterogeneity in process by state, but, otherwise, the same rates were used through time and across taxa. Future extensions of this work could examine additional sources of heterogeneity (e.g. variation in rates across time periods or across clades) [70], although such an increase in model complexity would require not only new methods, but also greater taxon sampling. The application of simulation approaches, as in this study and others [71], will be crucial for determining the power to estimate models and test macroevolutionary hypotheses.

## Supplementary Material

Supplemental Material

## Supplementary Material

Workflow

## Data accessibility

Data and scripts are available in Dryad at http://dx.doi.org/10.5061/dryad.486c0. Correspondence and requests for materials should be addressed to B.C.O. (bomeara@utk.edu) or S.D.S (stacey.d.smith@colorado.edu).
