# Supplementary material for: Non-equilibrium dynamics and floral trait interactions shape extant angiosperm diversity
Source: Proc Biol Sci. 2016 May 11;283(1830):20152304. doi: 10.1098/rspb.2015.2304 (PMC4874697; doi:10.1098/rspb.2015.2304)
Supplement: Supplemental Material [file rspb20152304supp1.pdf]

Table S1. Character coding scheme. The term perianth refers to the two outer whorls (calyx and corolla) and the term tepals applies when the two whorls are not differentiated. Our character polarity (ancestral vs. derived) follows Stebbin's original (1951) analysis although the states vary slightly because we favored grouping states that were functionally similar. For example, bisymmetry was combined with radial symmetry because pollinators can approach and interact with both types of flowers from multiple directions. These definitions were applied to score taxa at the species level although family or genus-level descriptions were used when the states were fixed within these groups.

Character	State 0 (Ancestral)	State 1 (Derived).
1. Perianth presence	Perianth (both sepals and petals or tepals) present	Corolla absent
2. Perianth fusion	Free (based on corolla or tepals if present, calyx if corolla was absent)	Fused (any amount of fusion is coded as fused)
3. Perianth symmetry	Radially symmetric (bisymmetric or polysymmetric; based on corolla or tepals if present, calyx if corolla absent)	Bilaterally symmetric or asymmetric
4. Fertile stamen number	Many (more than twice as many as merosity; staminodes were not considered)	Few (equal to or less than twice merosity)
5. Carpel fusion	Free or a single carpel	Fused (includes a single carpel)
6. Ovary position	Superior (includes hypanthia)	Inferior (includes partially inferior and half-inferior)

Table S2. Calibration and constraints for dating analysis. Calibrated nodes, identified as the most recent common ancestor (MRCA) of two terminal taxa, and the stem group (SG) or crown group (CG) of the clade they represent are indicated. The calibration age provided by each fossil was obtained from the uppermost (youngest) boundary to which the fossil is assigned, based on Gradstein and Ogg (2004). Calibrations were implemented as fixed points (fixed), minimum (min) or maximum (max) ages.

MRCA	CLADE	FOSSIL AGE (Ma)	CONSTRAINT	FOSSIL	REFERENCE
1. *Amborella trichopoda, Romanoa tamnoides*	CG Angiospermae	136	fixed	Tectate-columellate, perforate to reticulate, inaperturate pollen grains	(*28-30*)
2. *Barclaya longifolia, Cabomba caroliniana*	CG (Cabombaceae + Nymphaeaceae)	112	min	*Monetianthus mirus*	(*31, 32*)
3. *Illicium henryi, Austrobaileya scandens*	CG Austrobaileyales	125	min	Seeds with epidermal cells with anticlinal undulate walls	(*33*)
4. *Chloranthus japonicas, Hedyosmum arborescens*	CG Chloranthales	125	min	Stamens with *Clavatipollenites* pollen; isolated stamens, staminate inflorescences and pistillate flowers with *Asteropollis* pollen	(*33*)
5. *Caladium bicolor, Wolffiella caudata*	CG Araceae	125	min	*Mayoa portugallica*	(*34*)
6. *Gaussia attenuata, Austrodanthonia mera*	CG Commelinidae	85.8	min	Palm pollen and leaves	(*33, 35, 36*)
7. *Elasis hirsute, Paramomum petaloideum*	SG Zingiberales	83.5	min	*Spirematospermum chandlerae*	(*37*)
8. *Leptocarpus similis, Austrodanthonia mera*	SG Poaceae, SG Restionaceae	65.5	min	Pollen of Poaceae; pollen of Restionaceae	(*38, 39*)
9. *Canella winterana, Pseudowintera axillaris*	SG Winteraceae	125	min	*Walkeripollis gabonensis*	(*40, 41*)
10. *Lactoris fernandeziana,*	SG Lactoridaceae	89.3	min	*Lactoripollenites africanus*	(*42*)

Aristolochia littoralis					
11. *Saururus chinensis, Peperomia lancifolia*	SG Saururaceae	33.9	min	*Saururus biloba*	(*43*)
12. *Pycnanthus angolensis, Pseuduvaria dielsiana*	CG Magnoliales	112	min	*Endressinia brasiliana*	(*44*)
13. *Magnolia tripetala, Pseuduvaria dielsiana*	SG Magnoliaceae	96.5	min	*Archaeanthus linnenbergeri*	(*45*)
14. *Siparuna decipiens, Sextonia pubescens*	SG Lauraceae	105.8	min	*Potomacanthus lobatus*	(*46, 47*)
15. *Ranunculus muricatus, Romanoa tamnoides*	CG Eudicotyledoneae	125	max	Tricolpate pollen grains	(*48, 49*)
16. *Antizoma angustifolia, Ranunculus muricatus*	SG Menispermaceae	65.5	min	Menispermaceae endocarps	(*33*)
17. *Platanus occidentalis, Nelumbo lutea*	SG Platanaceae	105.8	min	*Platanocarpus brookensis*, *Aquia brookensis*, *Sapindopsis*	(*50*)
18. *Corylopsis sinensis, Jepsonia parryi*	CG Saxifragales	89.3	min	*Microaltingia apocarpela*	(*51*)
19. *Salvadora persica, Arabis alpina*	SG (Capparidaceae + Brassicaceae))	89.3	min	*Dressiantha bicarpellata*	(*52*)
20. *Bursera crenata, Salvadora persica*	SG Sapindales	55.8	min	*Acer* sp., *Dipteronia* sp., *Koelreuteria* sp.	(*53*)
21. *Bursera crenata, Tetragastris panamensis*	CG Burseraceae	51	min	*Bursericarpum aldwickense*	(*54, 55*)
22. *Citropsis schweinfurthii, Acer triflorum*	SG Rutaceae	33.9	min	*Euodia* sp., *Rutaspermum* sp., *Ptelea* sp.	(*53*)
23. *Ceiba pentandra, Schoutenia glomerata*	CG Malvaceae	33.9	min	*Craigia* sp., *Tilia* sp.	(*53*)
24. *Combretum molle, Microlicia fasciculata*	SG Combretaceae	87.5	min	*Esgueiria futabensis*	(*56*)
25. *Dapania racemosa, Tetratheca setigera*	SG Cunoniaceae	83.5	min	*Platydiscus peltatus*	(*57*)

26. *Trichocarya splendens, Romanoa tamnoides*	CG Malpighiales	89.3	min	*Paleoclusia chevalieri*	(*58*)
27. *Muraltia stipulacea, Pilea quercifolia*	SG Fagales	93.5	min	Normapolles pollen	(*59-61*)
28. *Senna mollissima, Muraltia stipulacea*	SG Fabaceae	55.8	min	Caesalpinioid fruits	(*62, 63*)
29. *Rosa luciae onoei, Sorbus aucuparia*	CG Rosaceae	37.2	min	*Prunus* sp., *Rosa* sp.	(*53*)
30. *Trymalium ledifolium, Sorbus aucuparia*	SG Rhamnaceae	48.6	min	*Pailurus* sp. (this fossil is supposed to calibrate SG Rhamnaceae)	(*53*)
31. *Rumex hastatus Limonium oblanceolatum*	SG Polygonaceae	5.33	min	Polygonaceae fruits	(*43, 64-66*)
32. *Phytolacca acinosa, Mirabilis alipes*	SG Phytolaccaceae	70.6	min	*Coahuilacarpon phytolaccoides*	(*67*)
33. *Cajophora macrocarpa, Coutoubea ramosa*	SG Cornales	89.3	min	*Tylerianthus crossmanensis*	(*68*)
34. *Impatiens rubrostriata, Astroloma ciliatum*	CG Ericales	89.3	min	*Paleoenkianthus sayrevillensis*	(*69*)
35. *Coutoubea ramose, Phryma leptostachya asiatica*	CG Garryidae (Lamiids)	83.5	min	*Scandianthus* spp.	(*37*)
36. *Geniostoma rupestre, Xysmalobium parviflorum*	SG Apocynaceae	37.2	min	Apocynaceae seeds	(*53*)
37. *Fraxinus mandshurica, Phryma leptostachya asiatica*	SG Oleaceae	33.9	min	*Fraxinus* sp.	(*53*)
38. *Incarvillea younghusbandii, Petrea racemosa*	SG Bignoniaceae	28.4	min	*Catalpa* sp.	(*53*)
39. *Scabiosa atropurpurea, Viburnum opulus sargentii*	CG Dipsacales	33.9	min	*Diplodipelta* sp.	(*70*)

40. *Pseudopanax laetevirens, Angelica tatianae*	CG Apiales	37.2	min	*Toricellia bonensii*	(*53*)

Table S3. Transition rate models for focal and non-focal states. K is the number of free parameters (transition rates) in the model. Each model contains up to four transition rates (q_{NF}, q_{NN}, q_{FF}, q_{FN}), where "N" denotes the non-focal state and "F" the focal state. The rate q_{NF} is thus the rate of transitions from the non-focal to the focal state.

	Model	K	q_{NF}	q_{NN}	q_{FF}	q_{FN}
1.	Equal	1	$q_{NF} = q_{NN} = q_{FF} = q_{FN}$			
2.	Inflow unique	2	q_{NF}	$q_{NN} = q_{FF} = q_{FN}$		
3.	Outflow unique	2	$q_{NF} = q_{NN} = q_{FF}$			q_{FN}
4.	Inflow and outflow different	3	q_{NF}	$q_{NN} = q_{FF}$		q_{FN}
5.	Free	4	q_{NF}	q_{NN}	q_{FF}	q_{FN}

Table S4. Diversification rate models for focal and non-focal states. K is the number of free parameters (rates) in the model. Each model contains up to four rates (λ_F, λ_N, μ_F, μ_N) where λ is the speciation rate, μ is the extinction rate, and "N" and "F" denote the non-focal and focal states, respectively.

	**Model**	**K speciation**	**K extinction**	$\lambda_{\boldsymbol{F}}$	$\lambda_{\boldsymbol{N}}$	$\mu_{\boldsymbol{F}}$	$\mu_{\boldsymbol{N}}$
1.	Yule	1	0	$\lambda_F = \lambda_N$		0	
2.	Two-rate Yule	2	0	λ_F	λ_N	0	
3.	Simple birth-death	1	1	$\lambda_F = \lambda_N$		$\mu_F = \mu_N$	
4.	Two birth, one death	2	1	λ_F	λ_N	$\mu_F = \mu_N$	
5.	One birth, two death	1	2	$\lambda_F = \lambda_N$		μ_F	μ_N
6.	Free	2	2	λ_F	λ_N	μ_F	μ_N

Table S5. Relative diversification rates from simulated replicate datasets. Columns show rates rates for the 8 possible combinations of binary states in the three focal traits (traits 1, 3, and 4; Table S2) relative to the rate of the focal combination (0x11xx). The states for these three characters follow Table S2, and the other characters (designated with an 'x') can be either state. Thus, 0x00xx represents the combination of petals present, radial symmetry, many stamens, and any state for the other three characters.

rep	div0x00xx	div0x01xx	div0x10xx	div0x11xx	div1x00xx	div1x01xx	div1x10xx	div1x11xx
1	0.51094	0.51094	0.93017	1.00000	0.51094	0.51094	0.92391	0.98432
2	0.99995	0.58739	0.99998	1.00000	0.99995	0.99995	0.99996	0.99997
3	0.99209	0.55577	0.99974	1.00000	0.99209	0.99209	0.99972	0.99998
4	0.61513	0.61477	0.76638	1.00000	0.61513	0.61513	0.75359	0.78563
5	0.61049	0.60540	0.83198	1.00000	0.61049	0.61049	0.82396	0.94185
6	0.74217	0.63433	0.74814	1.00000	0.74217	0.74217	0.74264	0.74323
7	0.99999	0.79545	1.00000	1.00000	0.99999	0.99999	1.00000	1.00000
8	0.99125	0.67779	0.99493	1.00000	0.99125	0.99125	0.99315	0.99585
9	0.57656	0.56687	0.92411	1.00000	0.57656	0.57656	0.92361	0.99634
10	0.55412	0.54387	0.55795	1.00000	0.55412	0.55412	0.55496	0.55506
11	0.56051	0.56047	0.60821	1.00000	0.56051	0.56051	0.60477	0.61020
12	0.99582	0.99435	0.99701	1.00000	0.99582	0.99582	0.99700	1.00000
13	1.00000	0.93789	1.00000	1.00000	1.00000	1.00000	1.00000	1.00000
14	0.99376	0.64125	0.99685	1.00000	0.99376	0.99376	0.99674	0.99773
15	0.96231	0.62904	0.99960	1.00000	0.96231	0.96231	0.99960	1.00000
16	0.60832	0.60832	0.67374	1.00000	0.60832	0.60832	0.60832	0.60832
17	0.61683	0.61139	0.71463	1.00000	0.61683	0.61683	0.71463	0.97035
18	0.73275	0.71438	0.96266	1.00000	0.73275	0.73275	0.91967	0.93751
19	0.59086	0.59085	0.59144	1.00000	0.59086	0.59086	0.59102	0.59106
20	0.97958	0.62811	0.98743	1.00000	0.97958	0.97958	0.98712	0.99916
21	0.59418	0.59416	0.67063	1.00000	0.59418	0.59418	0.62078	0.62136
22	0.61136	0.61135	0.95051	1.00000	0.61136	0.61136	0.94875	0.99670

Figure S1. The highly skewed distribution of combinations of six floral characters among the 464 angiosperm species sampled for this analysis. Character states (ancestral in black and derived in purple) are indicated for each combination. Height of a bar indicates the frequency of the character-state combination. Light grey area represents the 95% CI of ordered histograms based on repeated random sampling from a multinomial distribution with equal frequencies for each combination.

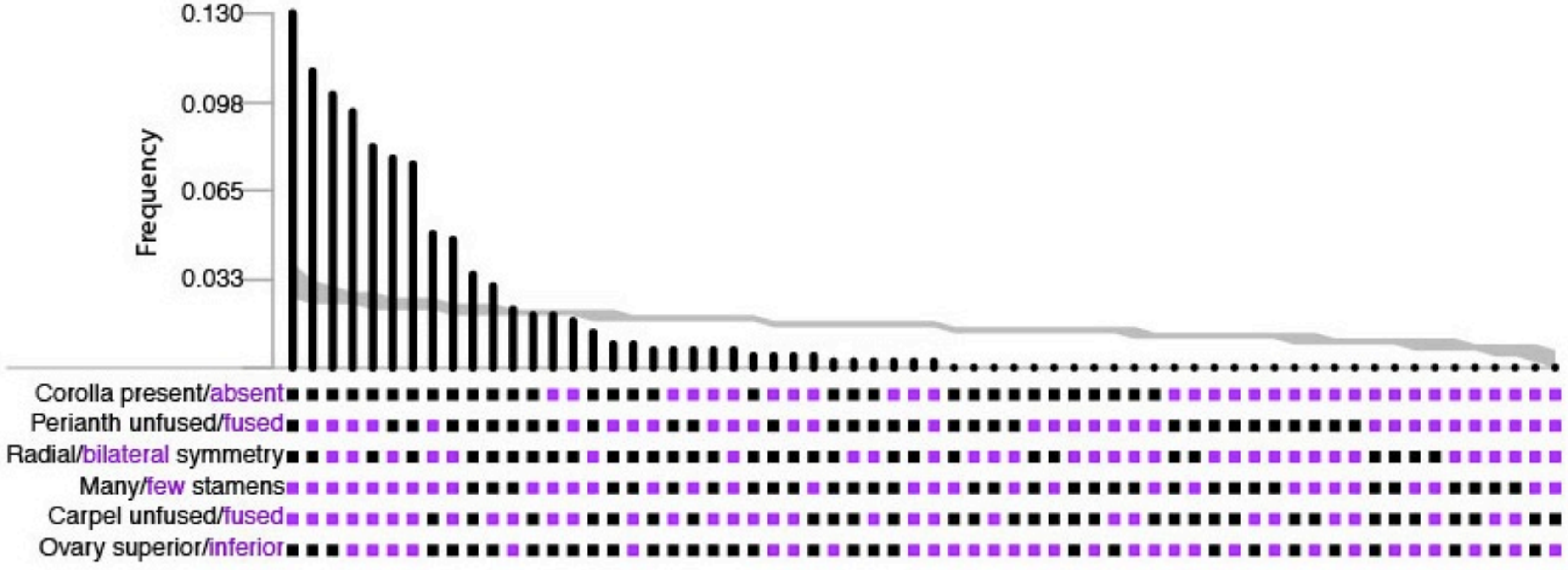

Figure S2. Results of parametric bootstrapping using model averaged diversification and transition rates. A) Diversification rates for the 8 possible combinations of binary states in the three focal traits (traits 1, 3, and 4; Table S2). The states for these three characters follow Table S1, and the other characters (designated with an 'x') can be either state. Each point represents the model-averaged value from one simulated dataset. The mean values from the 22 simulated datasets are shown with a purple bar and the values from the original dataset are shown with a red bar. B) Transition rates among the character states. Only rates involving the three focal traits are shown. Symbols follow A).

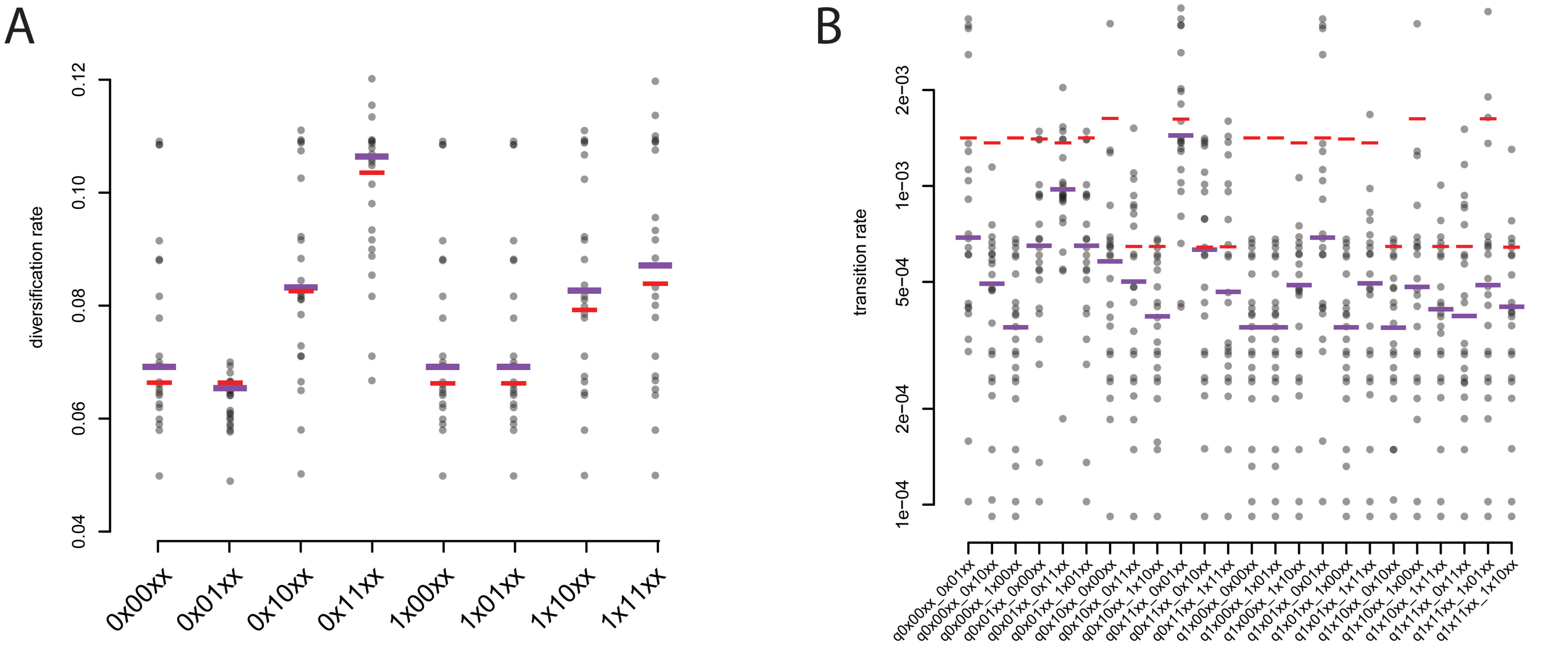

REFERENCES

1. S. A. Smith, J. M. Beaulieu, M. J. Donoghue, *Bmc Evol Biol* **9**, (Feb 11, 2009).
2. M. W. Chase *et al.*, *Annals of the Missouri Botanical Garden* **80**, 528 (1993).
3. D. E. Soltis *et al.*, *Botanical Journal of the Linnean Society* **133**, 381 (Aug, 2000).
4. Y. L. Qiu *et al.*, *Nature* **402**, 404 (Nov 25, 1999).
5. A. Stamatakis, *Bioinformatics* **22**, 2688 (Nov 1, 2006).
6. S. A. Smith, B. C. O'Meara, *Bioinformatics* **28**, 2689 (Oct 15, 2012).
7. S. A. Magallon, M. J. Sanderson, *Evolution* **59**, 1653 (Aug, 2005).
8. A. Stamatakis, P. Hoover, J. Rougemont, *Syst Biol* **57**, 758 (2008).
9. G. L. Stebbins, *Evolution* **5**, 299 (1951).
10. P. K. Endress, *Taxon* **31**, 48 (1982).
11. K. Faegri, L. van der Pijl, *The principles of pollination ecology*. (Pergamon Press, Oxford, 1979).
12. H. Citerne, F. Jabbour, S. Nadot, C. Damerval, *Adv Bot Res* **54**, 85 (2010).
13. W. S. Armbruster, C. Pelabon, T. F. Hansen, G. H. Bolstad, *New Phytol* **183**, 600 (2009).
14. D. L. Mulcahy, *Science* **206**, 20 (1979).
15. W. S. Armbruster, E. M. Debevec, M. F. Willson, *J Evolution Biol* **15**, 657 (Jul, 2002).
16. V. Grant, *Evolution* **4**, 179 (1950).
17. J. N. Thompson, O. Pellmyr, *Ecology* **73**, 1780 (Oct, 1992).
18. P. K. Endress, J. A. Doyle, *Am J Bot* **96**, 22 (Jan, 2009).
19. P. K. Endress, *Ann Bot-London* **107**, 1465 (Jun, 2011).
20. C. E. Bessey, *Annals of the Missouri Botanical Garden* **2**, 109 (1915).
21. A. Cronquist, *The evolution and classification of flowering plants*. (The New York Botanical Garden, Bronx, NY, 1988).
22. A. Takhtajan, *Diversity and classification of flowering plants*. (Columbia University Press, New York, NY, 1997).
23. W. P. Maddison, *Evolution* **60**, 1743 (Aug, 2006).
24. R. G. FitzJohn, W. P. Maddison, S. P. Otto, *Syst Biol* **58**, 595 (Dec, 2009).
25. M. Pagel, *P Roy Soc Lond B Bio* **255**, 37 (Jan 22, 1994).
26. B. C. O'Meara, *Annual Review of Ecology, Evolution, and Systematics* **43**, 267 (2012).
27. H. Akaike, *Ieee T Automat Contr* **Ac19**, 716 (1974).
28. N. F. Hughes, A. B. Mcdougall, *Rev Palaeobot Palyno* **50**, 255 (Feb, 1987).
29. N. F. Hughes, A. B. Mcdougall, J. L. Chapman, *Journal of micropaleontology* **10**, 75 (1991).
30. G. J. Brenner, in *Flowering plant origin, evolution, and phylogeny,* D. W. Taylor, L. J. Hickey, Eds. (Chapman and Hall, New York, 1996), pp. 91-115.
31. E. M. Friis, K. R. Pedersen, P. R. Crane, *Nature* **410**, 357 (Mar 15, 2001).
32. E. M. Friis, K. R. Pedersen, M. von Balthazar, G. W. Grimm, P. R. Crane, *International Journal of Plant Sciences* **170**, 1086 (Oct, 2009).
33. E. M. Friis, K. R. Pedersen, P. R. Crane, *Palaeogeogr Palaeocl* **232**, 251 (Mar 22, 2006).

34. E. M. Friis, K. R. Pedersen, P. R. Crane, *P Natl Acad Sci USA* **101**, 16565 (Nov 23, 2004).
35. R. A. Christopher, *Palynology* **3**, 73 (1979).
36. C. P. Daghlian, *Bot Rev* **47**, 517 (1981).
37. E. M. Friis, A. Skarby, *Ann Bot-London* **50**, 569 (1982).
38. H. P. Linder, *Kew Bulletin* **42**, 297 (1987).
39. D. M. Jarzen, *Palynology* **2**, 29 (1978).
40. J. A. Doyle, *Annals of the Missouri Botanical Garden* **87**, 303 (2000).
41. J. A. Doyle, C. L. Hotton, J. V. Ward, *Am J Bot* **77**, 1558 (Dec, 1990).
42. D. H. Mai, *Flora* **176**, 449 (1995).
43. E. M. Friis, *Biol Skr Dan Vid Sel* **24**, 1 (1985).
44. B. A. R. Mohr, M. E. C. Bernardes-de-Oliveira, *International Journal of Plant Sciences* **165**, 1121 (Nov, 2004).
45. D. L. Dilcher, P. R. Crane, *Annals of the Missouri Botanical Garden* **71**, 351 (1984).
46. P. R. Crane, E. M. Friis, K. R. Pedersen, *Plant Syst Evol*, 51 (1994).
47. M. von Balthazar, K. R. Pedersen, P. R. Crane, M. Stampanoni, E. M. Friis, *Am J Bot* **94**, 2041 (Dec, 2007).
48. N. F. Hughes, A. B. Mcdougall, *Rev Palaeobot Palyno* **65**, 145 (Oct 30, 1990).
49. J. A. Doyle, *Cretaceous Res* **13**, 337 (Aug, 1992).
50. P. R. Crane, K. R. Pedersen, E. M. Friis, A. N. Drinnan, *Syst Bot* **18**, 328 (Apr-Jun, 1993).
51. Z. K. Zhou, W. L. Crepet, K. C. Nixon, *Am J Bot* **88**, 753 (May, 2001).
52. M. A. Gandolfo, K. C. Nixon, W. L. Crepet, *Am J Bot* **85**, 964 (Jul, 1998).
53. S. R. Manchester, *Annals of the Missouri Botanical Garden* **86**, 472 (1999).
54. E. M. Reid, M. E. J. Chandler, *The London Clay Flora*. (British Museum (Natural History), London, 1933).
55. M. Collinson, *Fossil plants of the London Clay*. Palaeontological Association Field Guides to Fossils (The Palaeontological Association, London, 1983), vol. 1.
56. M. Takahashi, P. R. Crane, H. Ando, *Paleontological Research* **3**, 81 (1999).
57. J. Schonenberger, E. M. Friis, M. L. Matthews, P. K. Endress, *Ann Bot-London* **88**, 423 (Sep, 2001).
58. W. L. Crepet, K. C. Nixon, *Am J Bot* **85**, 1122 (Aug, 1998).
59. D. J. Batten, *Rev Palaeobot Palyno* **35**, 125 (1981).
60. M. Kedves, in *Evolution, systematics, and fossil history of the Hamamelidae,* P. R. Crane, S. Blackmore, Eds. (Clarendon Press, Oxford, 1989), vol. 1-7.
61. B. Pacltova, *Palaeobotanist* **15**, 52 (1966).
62. P. S. Herendeen, P. R. Crane, in *Advances in legume systematics IV: the fossil record,* P. S. Herendeen, D. L. Dilcher, Eds. (Royal Botanic Gardens, Kew, 1992), vol. IV, pp. 57-68.
63. M. Lavin, P. S. Herendeen, M. F. Wojciechowski, *Syst Biol* **54**, 575 (Aug, 2005).
64. H.-J. Gregor, *Die Jungarten floren Suddeutschlands*. (Ferdinand Enke, Stuttgart, 1982).
65. J. Van der Burgh, *Rev Palaeobot Palyno* **52**, 299 (1987).
66. P. I. Dorofeev, *Miocene floras of the Tambov district*. (Akademii Nauk, Leningrad, 1988).
67. S. R. S. Cevallos-Ferriz, E. Estrada-Ruiz, B. R. Perez-Hernandez, *Am J Bot* **95**, 77 (Jan, 2008).

68. M. A. Gandolfo, K. C. Nixon, W. L. Crepet, *Am J Bot* **85**, 376 (Mar, 1998).
69. K. C. Nixon, W. L. Crepet, *Am J Bot* **80**, 616 (Jun, 1993).
70. S. R. Manchester, M. J. Donoghue, *International Journal of Plant Sciences* **156**, 709 (Sep, 1995).
