# Supplementary material for: Non-equilibrium dynamics and floral trait interactions shape extant angiosperm diversity
Source: Proc Biol Sci. 2016 May 11;283(1830):20152304. doi: 10.1098/rspb.2015.2304 (PMC4874697; doi:10.1098/rspb.2015.2304)

Workflow for creating simplified MuSSE models for bipartitions of character state space

This document describes how we estimated transitions rates and diversification rates across all combinations of six binary floral characters on diversification. The overall approach was to treat the state space as a network and examine transitions between sets of combinations (bipartitions of the network) as well as diversification within each set. We begin by considering one example set from all the possible combinations.

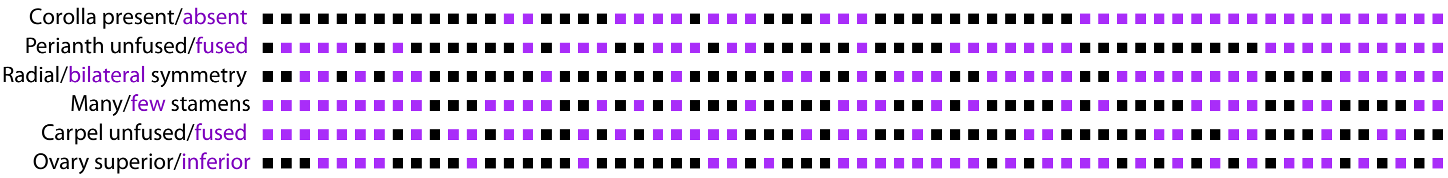

Each of the six traits (left) has an ancestral (black) and a derived state (purple). The columns of squares to the right represent the possible combinations of states; for example, the first column represents corolla present, perianth unfused, radial symmetry, few stamens, fused carpel, and superior ovary.

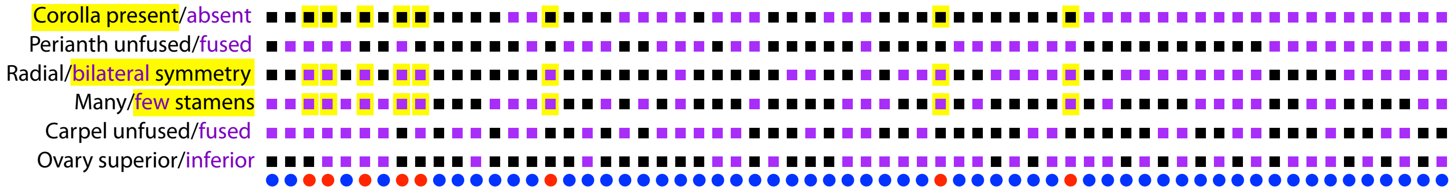

We identify a combination of states (in yellow) that might have a different transition and/or diversification rates than the other combinations. In this example the focal set of character states is corolla present, bilateral symmetry, and few stamens. We could move through each of the $2^6 = 64$ possible combinations of the six binary traits, and identify which fall in this set (those columns with a red dot below them).

These combinations can be viewed as a subset of the network of all the combinations. The figure below portrays the 64 combinations as network where those with corolla present, bilateral symmetry, and few stamens are in red. The characters are coded as strings of binary (0/1) variables, thus this set shares a zero (ancestral state of corolla present) for the first number (first character), a one (derived state of bilateral symmetry) for the third number (third character), and a one (derived state of few stamens) for the fourth number (fourth character). You may note that all edges in the network represent a change in the state of just one trait (000000 to 001000, for example). In theory in a full MuSSE model, each node could have its own unique speciation and extinction rate, and the rate of moving from one node to another node could have its own unique rate. For 64 states, this full MuSSE model would comprise 512 parameters (see text).

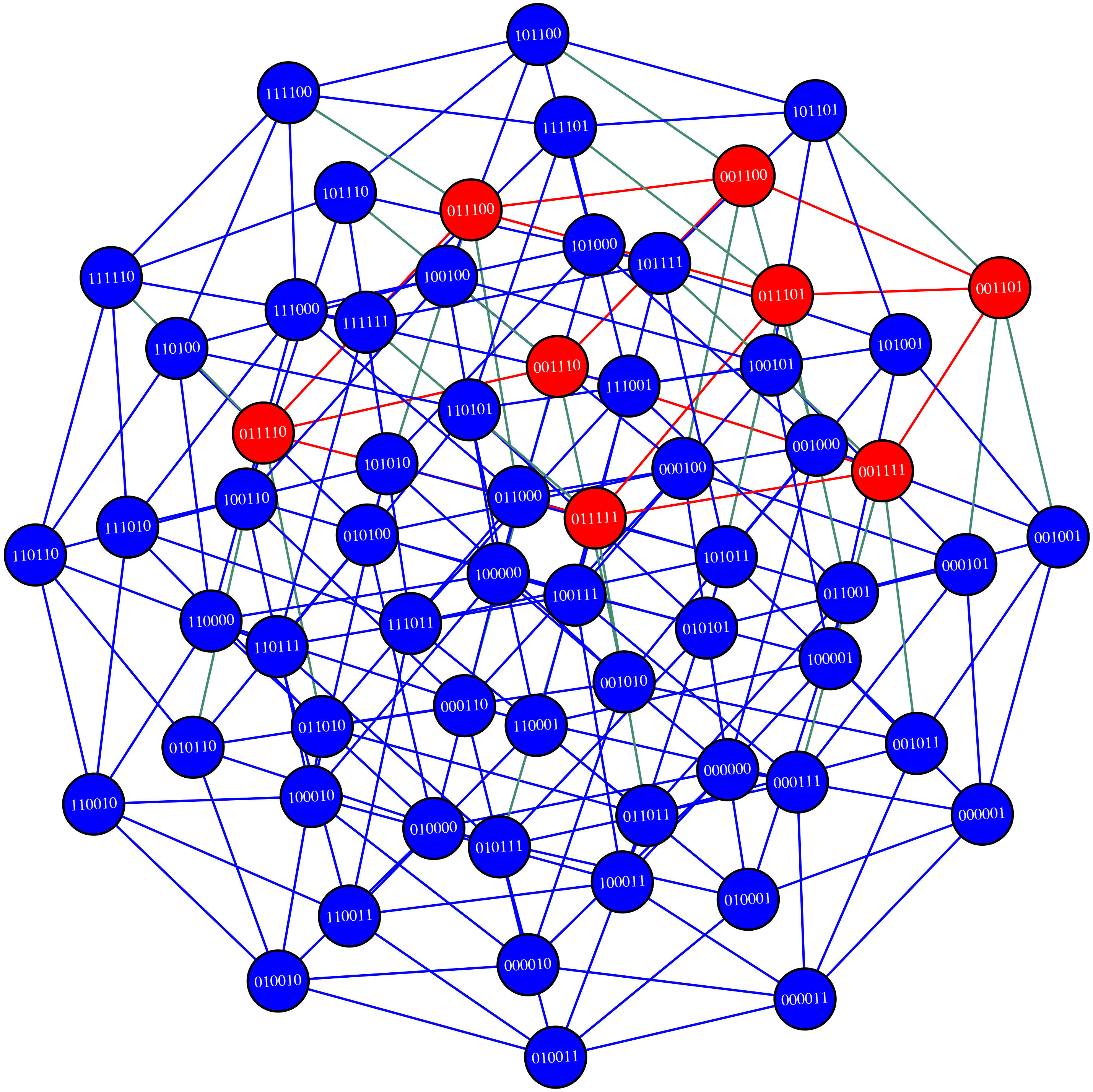

We simplify this complex model by fitting simpler models for bipartition of the network (sets of character combinations). In this figure, we have ‘pulled’ the set away from the rest of the network to visualize the bipartition. Red and blue lines connect nodes within the sets while green lines connect nodes between the sets.

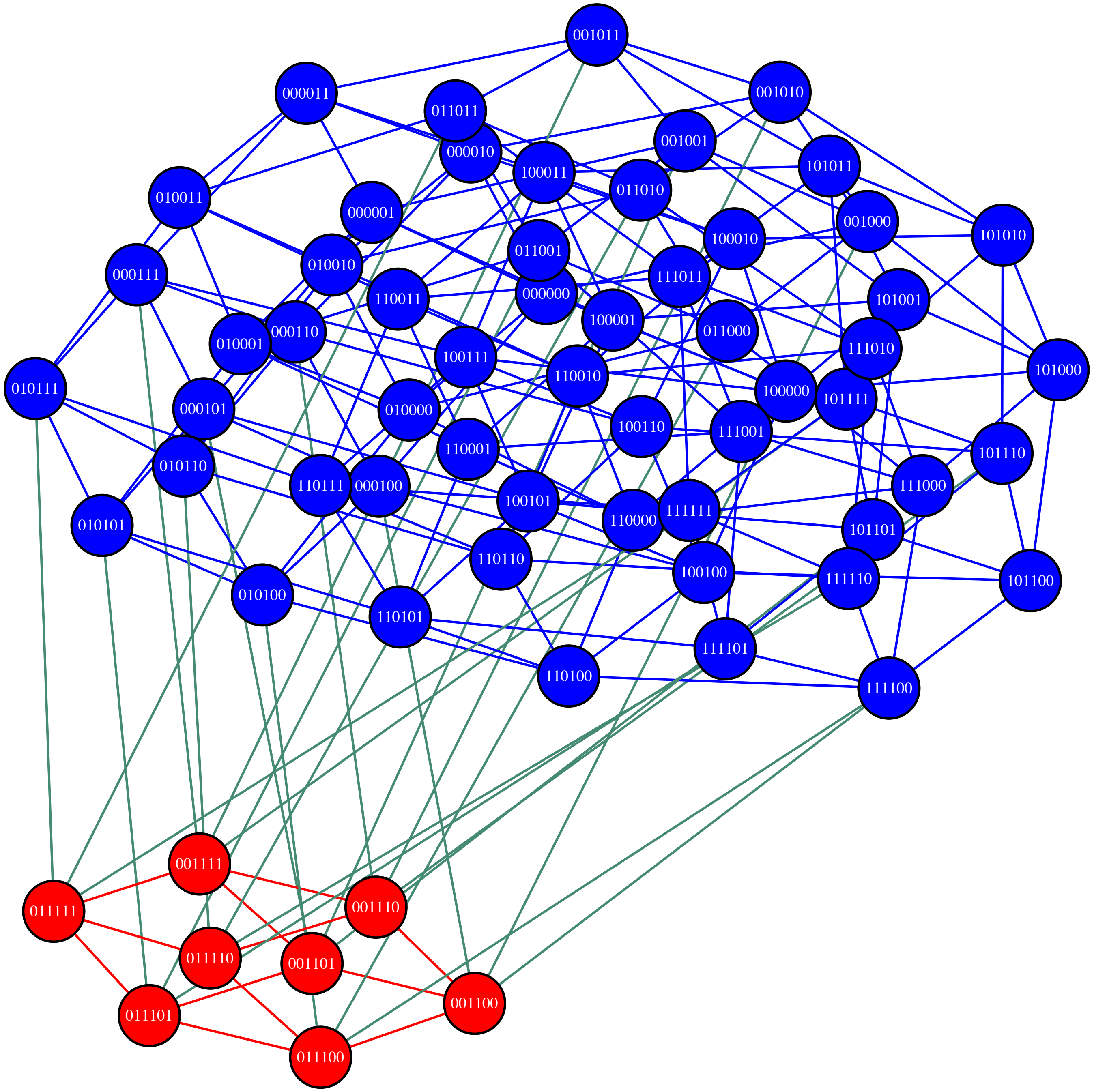

With such a bipartition, a simple model could be two allow just two speciation rates, one for the red nodes (which include all six character combinations that in contain our specified set of three states), one for the blue nodes, and the same for extinction rates. We could also imagine lumping the transition rates: one rate between the red nodes (the red lines), one rate between the blue nodes (blue lines), and rates from red to blue or from blue to red (green lines).

Birth_A

Death_A

Birth_B

Death_B

$\text{Transition}_{A \rightarrow A}$

$\text{Transition}_{B \rightarrow B}$

$\text{Transition}_{A \rightarrow B}$

$\text{Transition}_{B \rightarrow A}$

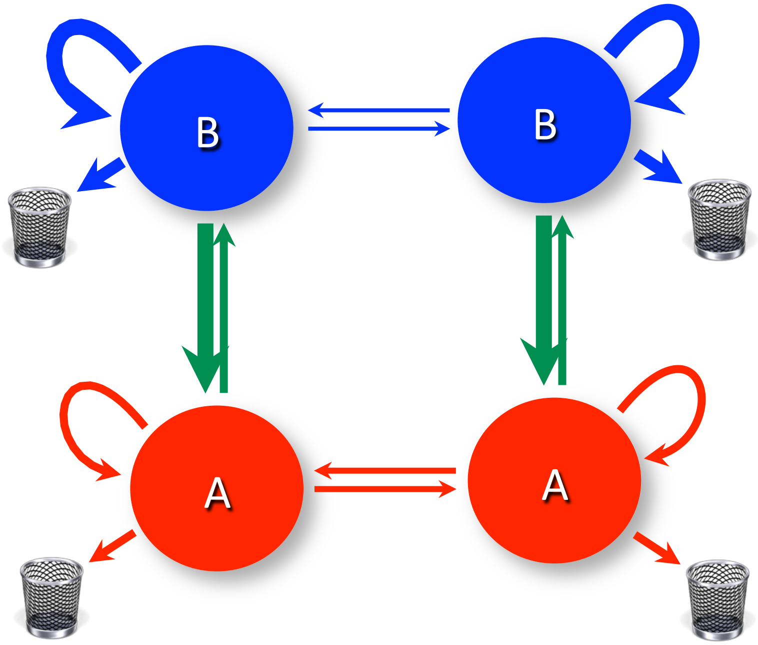

Thus, a complex model of potentially hundreds of rates gets reduced down to a fairly simple MuSSE model. We could then simplify it further: try models that have the death rates in A and B set to be the same, for example. Our analysis included thirty such models for each bipartition of the network, calculating the likelihood of the tree and data each time using diversitree.

This process of creating a bipartition and fitting 30 simple but realistic models can then be repeated across all possible partitions (all possible sets of character combinations). Three such examples are shown below (e.g., a set that is all taxa with zero for the character 1, etc.) Because the underlying data are the same, the AIC scores can be compared directly for all bipartitions and models. We can then identify the best fitting models in terms of division of the network into sets and in terms of diversification and transition rates. This allows us to determine which individual characters and character combinations experience differential diversification rates and how transition rates vary between the parts of the network.

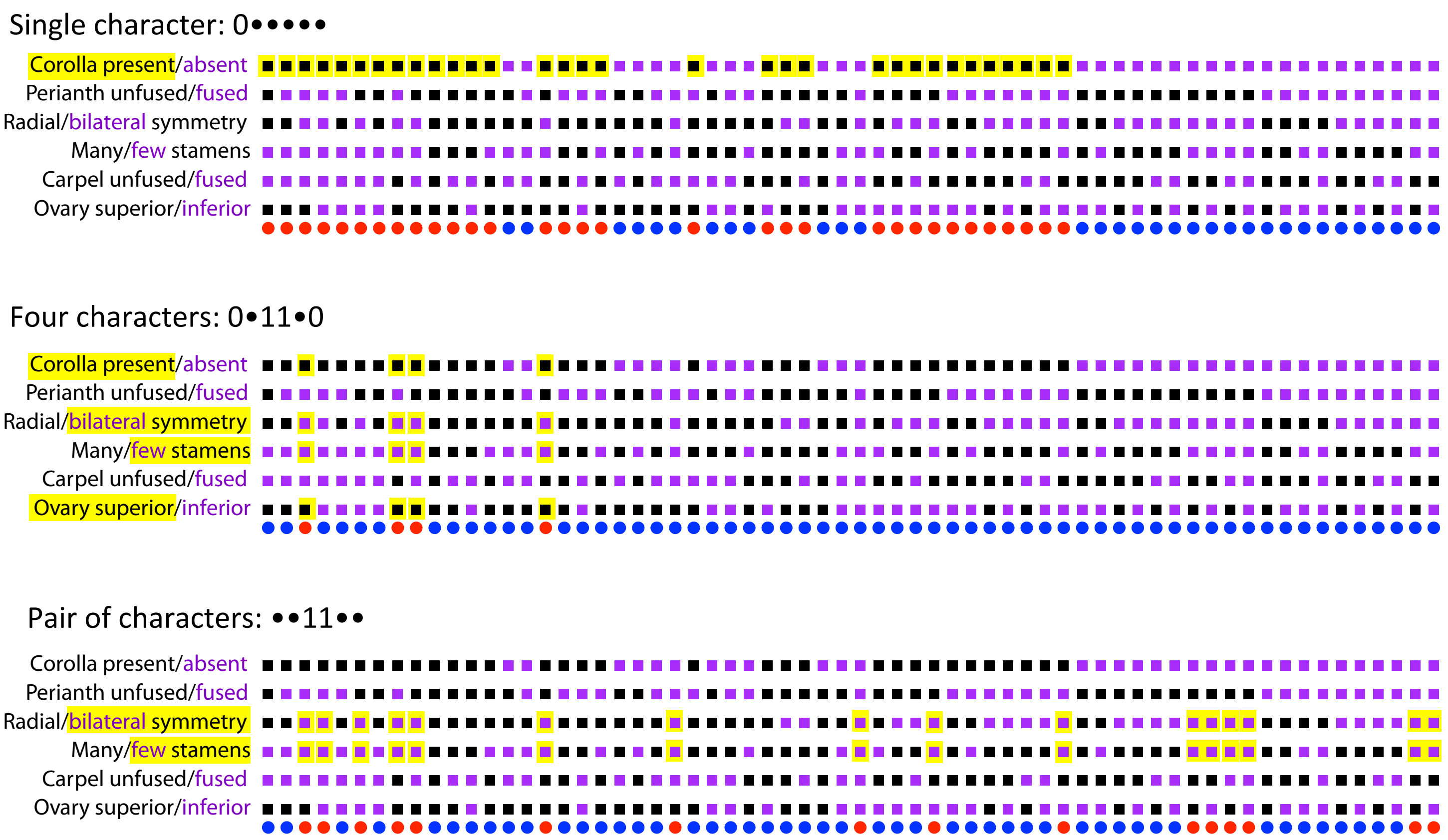

Supplement: Workflow [file rspb20152304supp2.pdf]
